# Two-Step Solvothermal Synthesis of (Zn_0.5_Co_0.5_Fe_2_O_4_/Mn_0.5_Ni_0.5_Fe_2_O_4_)@C-MWCNTs Hybrid with Enhanced Low Frequency Microwave Absorbing Performance

**DOI:** 10.3390/nano9111601

**Published:** 2019-11-11

**Authors:** Pengfei Yin, Limin Zhang, Hongjing Wu, Xing Feng, Jian Wang, Hanbing Rao, Yanying Wang, Jianwu Dai, Yuting Tang

**Affiliations:** 1 College of Science, Sichuan Agricultural University, Ya’an 625014, China; 12341@sicau.edu.cn (X.F.); 10902@sicau.edu.cn (J.W.);; 2 Key Laboratory of Space Applied Physics and Chemistry (Ministry of Education), School of Science, Northwestern Polytechnical University, Xi’an 710072, China; liminzhang@nwpu.edu.cn; 3 College of Mechanical and Electrical Engineering, Sichuan Agricultural University, Ya’an 625014, China; jwdai@sicau.edu.cn

**Keywords:** Zinc cobalt ferrite, manganese nickel ferrite, multi-walled carbon nanotubes, microwave absorbing property, low frequency band

## Abstract

In this study, the quaternary hybrid of (Zn_0.5_Co_0.5_Fe_2_O_4_/Mn_0.5_Ni_0.5_Fe_2_O_4_)@C-MWCNTs with high-performance in low frequency electromagnetic absorption was synthesized via a facile two-step solvothermal synthesis method. The physicochemical properties as well as electromagnetic parameters and microwave absorption performance were characterized by XRD, SEM, TEM, RS, TGA, and VNA, respectively. The results indicate a nuclear-shell morphology of this hybrid for amorphous carbon coated on the surface of Zn_0.5_Co_0.5_Fe_2_O_4_ and Mn_0.5_Ni_0.5_Fe_2_O_4_ mixed polycrystalline ferrites. In addition, the MWCNTs synchronously enwind in the nuclear-shell NPs to form a special cross-linking structure. The outstanding low frequency microwave absorption property is attributed to the synergistic effect of dielectric and magnetic loss, better impedance matching condition, and excellent attenuation characteristics of the as-prepared paramagnetic quaternary hybrid. Maximum *RL* of −35.14 dB at 0.56 GHz with an effective absorption bandwidth in the range of 0.27–1.01 GHz can be obtained with thickness of 5 mm. This hybrid exhibits superior low frequency microwave absorption properties compared with other ferrite-carbon nanocomposites. This investigation provides a new route to prepare suitable candidates for the absorption of electromagnetic waves in a low frequency band on account of its good performance and simple preparation process.

## 1. Introduction

The rapid development of detection and communication technologies in the military and commercial fields has resulted in more attention being paid to electromagnetic interference (EMI), electromagnetic pollution, and radar stealth. The creation of microwave absorption materials (MAMs) with good performance is an effective approach to address these problems [1,2,3,4]. It is well known that MAMs can attenuate electromagnetic waves by converting electromagnetic energy into heat energy or by dissipating it via all kinds of loss mechanisms. At this stage, MAMs have already been extensively investigated; they include carbon materials [5,6,7], conducting polymers [8,9,10], ceramic materials [11,12,13], magnetic metals and alloys [14,15,16], ferrites [17,18,19], etc. Among these materials, ferrites are widely used as MAMs for their advantages of high Curie temperature, special magnetic property, good chemical and thermo-stability, and low costs [20]. However, pure magnetic ferrites are not sufficient as efficient microwave absorbers due to the unilateral magnetic loss mechanism resulting in a poor impedance matching condition. Generally, the ideal MAMs require strong absorption ability and broad absorbing bandwidth; thus, magnetic ferrites can be modified or composited with other MAMs to optimize their electromagnetic absorption performance. For instance, J. Xiang et al. [21] prepared Ni_0.4_Co_0.2_Zn_0.4_Fe_2_O_4_/BaTiO_3_ fibers via a simple electrospinning and subsequent heat treatment; the maximum reflection loss (*RL*) value reached −65.6 dB at 15.7 GHz with a broad effective absorption bandwidth below −10 dB of 7.8 GHz for a layer thickness of 5 mm. M.M. Ismail et al. [22] synthesized CoFe_2_O_4_/PANI-PTSA composite by sol-gel method; the as-prepared composite presented a wider absorption range and maximum *RL* was −28.4 dB at 8.1 GHz. Q. Chen et al. [23] fabricated the CIP@SiO_2_@Mn_0.6_Zn_0.4_Fe_2_O_4_ composite by using chemical co-precipitation technology; the optimal *RL* value of the composite with thickness of 2 mm was able to reach up to −44.24 dB at 11.57 GHz as well as bandwidth of *RL* < −10 dB from 9.04 to 16.16 GHz. S. Acharya et al. [24] prepared a flexible microwave absorber composite by using reduced graphene oxide, strontium ferrite, and poly methyl methacrylate matrix via a simple gel casting technique and in situ polymerization method; more than 99% absorption efficiency was achieved with a suitable combination of dielectric and magnetic coupling in X-band. 

In spite of this, present investigations on MAMs are mainly focused in the frequency range of 2–18 GHz. The electromagnetic absorption of these composites is very limited in the low frequency range of 0.1–2 GHz. However, the electromagnetic radiation generated from electronic devices used daily is in a low frequency range, and the detection frequency of many radars e.g. meter-wave radar, phased array radar, spaceborne radar, etc. has been extended to the low frequency region. Hence, this poses a significant challenge in the design of MAMs with outstanding electromagnetic wave absorption capacity in the low frequency band [25,26]. In recent years, L. Zhu et al. [27], K. Zhang et al. [28], and Y. Shao et al. [29] combined Fe_3_O_4_ NPs with multi-walled carbon nanotubes (MWCNTs) to enhance the electromagnetic absorption of ferrites for excellent dielectric loss, special one-dimensional nanostructure, low density and resistivity, and good thermal stability of MWCNTs. Moreover, the electromagnetic wave absorption of the composite in a low frequency band can also be heightened with the doping of MWCNTs [30]. 

Therefore, in the present work, the (Zn_0.5_Co_0.5_Fe_2_O_4_/Mn_0.5_Ni_0.5_Fe_2_O_4_)@C-MWCNTs (denoted as (ZCFO/MNFO)@C-MWCNTs henceforth) hybrid was synthesized via a two-step solvothermal method. The phase analysis, micro- and crystal structures, elemental distribution, Raman spectrum, thermogravimetry properties, electromagnetic parameters, and electromagnetic absorption performance of the as-prepared hybrid were characterized by X-Ray diffraction (XRD), scanning electron microscope (SEM), transmission electron microscope (TEM), Raman spectroscopy (RS), thermogravimetric analysis (TGA), and vector network analyzer (VNA) respectively. The intrinsic mechanisms of microwave absorption were also discussed. The results show that the quaternary hybrid can have excellent electromagnetic wave absorbing performance in a low frequency range, and such a hybrid can be used as a promising electromagnetic wave absorber in this band. 

## 2. Experiment

### 2.1. Materials

Zinc chloride (ZnCl_2_), cobalt chloride hexahydrate (CoCl_2_·6H_2_O), ferric chloride hexahydrate (FeCl_3_·6H_2_O), polyethylene glycol-6000, and sodium acetate trihydrate (NaAc) were purchased from Kelong Chemical Reagent Co. Ltd, Chengdu, China. The nickel chloride hexahydrate (NiCl_2_·6H_2_O) was purchased from Basf Biotechnology Co. Ltd, Hefei, China. The ethylene glycol and manganese chloride tetrahydrate (MnCl_2_·4H_2_O) were purchased from Sinopharm Chemical Reagent Co. Ltd, Shanghai, China. The multi-walled carbon nanotubes were purchased from Xianfeng Nano Technology Co. Ltd, Shuzhou, China. 

### 2.2. Preparation of Zn_0.5_Co_0.5_Fe_2_O_4_ NPs, Zn_0.5_Co_0.5_Fe_2_O_4_/Mn_0.5_Ni_0.5_Fe_2_O_4_ and (Zn_0.5_Co_0.5_Fe_2_O_4_/Mn_0.5_Ni_0.5_Fe_2_O_4_)@C-MWCNTs hybrids

The Zn_0.5_Co_0.5_Fe_2_O_4_ nanoparticles (denoted as ZCFO NPs henceforth) were prepared via the solvothermal synthesis method [31]. In detail, 0.85 g zinc chloride, 1.4875 g cobalt chloride hexahydrate, and 6.75 g ferric chloride hexahydrate were consecutively dissolved in 200 mL ethylene glycol. Then, 18 g sodium acetate trihydrate and 5 g polyethylene glycol-6000 were successively added into the previous solution with ultrasonic dispersion for 30 min. Afterwards, the obtained solution was sealed in a 300 mL Teflon-lined stainless-steel autoclave with temperature of 160 °C maintained for 6 h. ZCFO NPs was separated from the mixed solution with a NbFeB magnet after the reaction, then washed with ethanol for several times to get rid of the residual solution, and dried at 50 °C for 6 h. 

The (Zn_0.5_Co_0.5_Fe_2_O_4_/Mn_0.5_Ni_0.5_Fe_2_O_4_)@C-MWCNTs hybrid was obtained via the solvothermal method once again, i.e. 1.2375 g manganese chloride tetrahydrate, 1.485 g nickel chloride hexahydrate, and 6.75 g ferric chloride hexahydrate were in order dissolved in 200 mL ethylene glycol. Subsequently, 18 g sodium acetate trihydrate, 5 g polyethylene glycol 6000, and 2.6292 g ZCFO NPs obtained from the previous process and 0.8025 g MWCNTs were added into the above solution with ultrasonic dispersion and persistent stirring for 30 min. Then, the mixed solution was sealed in a Teflon-lined stainless-steel autoclave and the reaction temperature was maintained at 200 °C for 12 h. Finally, the as-prepared product was separated with a magnet and washed with ethanol, after which it was dried at 50 °C for 6 h. MWCNTs content in the as-prepared nanocomposite was ~12 wt%, which was decided as an optimal proportion according to our previous investigations and can be calculated by the mole ratio of reactants [32]. In addition, the preparation of Zn_0.5_Co_0.5_Fe_2_O_4_/Mn_0.5_Ni_0.5_Fe_2_O_4_ hybrid (denoted as ZCFO/MNFO henceforth) was done following the same process, without the addition of MWCNTs.

### 2.3. Characterization

The crystal structures of hybrids were analyzed by an X-ray diffraction system with Cu-*K*_α_ radiation of *λ* = 0.154 nm and scan step of 0.02° (D8-ADVANCE, Bruker Inc., Karlsruhe, German). The superficial micro-morphology of hybrids was characterized by a Scanning Electron Microscope (SEM, SU8100, Hitachi Inc., Tokyo, Japan). The transmission morphology, Selected Area Electron Diffraction (SAED), and elemental distribution were characterized by a Transmission Electron Microscope (TEM, Tecnai G2 F30, FEI Inc., Hillsboro, OR, USA). Raman spectra was measured with a Raman spectroscopy (In Via Reflex) by using a laser wavelength of 532 nm. Data of Thermogravimetric Analysis (TGA) was collected in air at a heating rate of 10 °C/min. The samples used for microwave absorption measurements were prepared by blending the products with paraffin in a mass percentage of 40 wt%; then the prepared samples were pressed into a toroidal shape with Φ_out_ = 7.00 mm, Φ_in_ = 3.04 mm, and different thicknesses of 2–5 mm. The S-parameters of S_11_ and S_21_ can be tested with coaxial transmission and reflection method by using the TD3618C Vector Network Analyzer (VNA, Tianda Co. Ltd, Chengdu, China). Thus, complex permittivity and permeability can be calculated on the basis of Nicolson and Ross’s theory [33].

## 3. Results and Discussion

**Figure 1:**
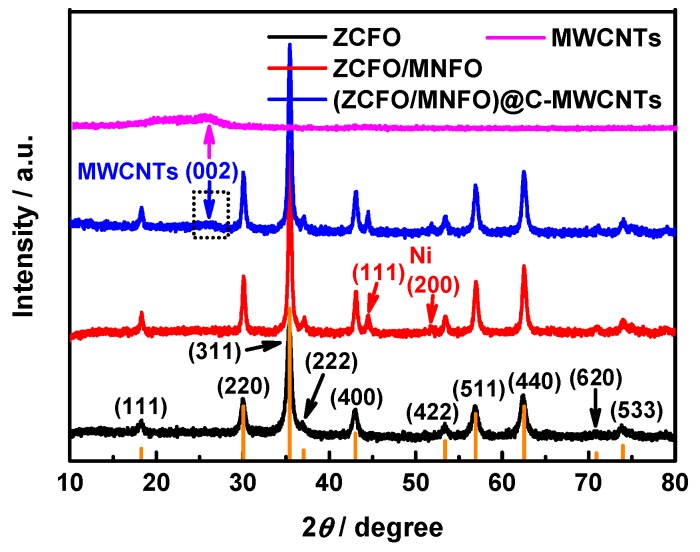
X-ray diffraction patterns of ZCFO, ZCFO/MNFO, and (ZCFO/MNFO)@C-MWCNTs hybrids.

The phase composition of the as-prepared hybrids has been confirmed by XRD; the patterns are illustrated in Figure 1. As estimated, the characteristic diffraction peaks of ZCFO at 2*θ* = 18.29°, 30.08°, 35.44°, 37.06°, 43.06°, 53.45°, 56.97°, 62.59°, 70.95°, and 74.01° are in good agreement with the (111), (220), (311), (222), (400), (422), (511), (440), (620), and (533) planes (JCPDS card no.22-1012). No other impurity peak was observed, confirming the spinel structure of ZCFO ferrite [34]. In addition, the crystal structure of MNFO was found to be quite similar to that of ZCFO; thus, the diffraction peaks of ZCFO/MNFO are almost entirely the same as ZCFO [35]. However, there are two weak impurity peaks at 2*θ* = 44.51° and 51.85°, assigned to the (111) and (200) planes of less Ni elementary substance (JCPDS card no.04-0850) reduced by ethylene glycol during the secondary hydrothermal reaction. Moreover, it shows that there exists a weak diffraction peak at 2*θ* = 26.38° corresponding to the (002) plane of MWCNTs (JCPDS card no.41-1487) in the quaternary (ZCFO/MNFO)@C-MWCNTs hybrid, indicating that the MWCNTs is composited well with ferrites. The intensity of this diffraction peak is much weaker than those of ferrites, as the wall thickness of MWCNTs used here is thin enough [36].

**Figure 2:**
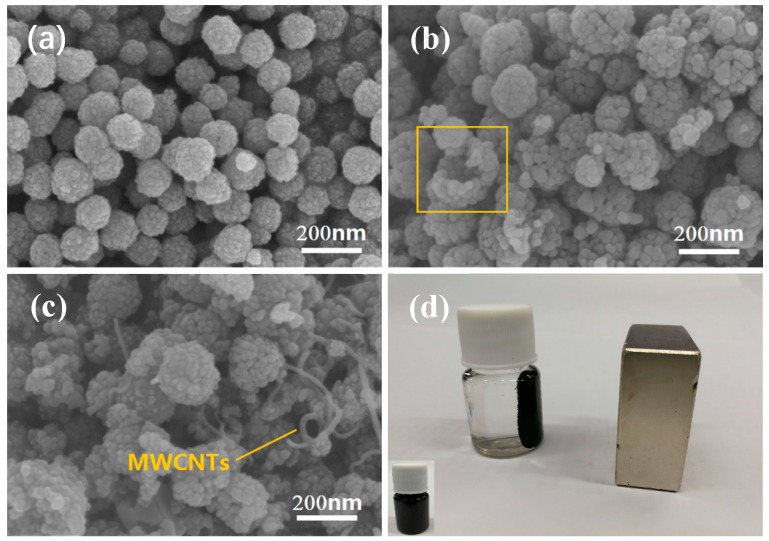
SEM images of ZCFO (**a**), ZCFO/MNFO (**b**), (ZCFO/MNFO)@C-MWCNTs (**c**), and macro-morphology of the (ZCFO/MNFO)@C-MWCNTs hybrid (**d**).

Figure 2 presents the scanning electron micrograph of the as-prepared hybrids. As shown in Figure 2a, the ZCFO ferrite exhibits ball-like rough NPs, the diameters of which are about ~120 nm. After the second solvothermal reaction, the MNFO ferrite is coated on the surface of ZCFO micro-grains to form the hybrid of ZCFO/MNFO; thus, the particle size of NPs become larger than that of the former. There also exists less-hollow NPs with many micro-grains, as shown in the square area of Figure 2b. Figure 2c shows the distribution of MWCNTs in the quaternary hybrid; it suggests that the MWCNTs are attached to the surface of the NPs and connect the NPs with each other, which is helpful for the conducting loss of micro-current generated from alternating electromagnetic waves. The cleaned quaternary hybrid can be evenly dispersed in the ethanol to form a black suspension liquid, as shown in the left bottom of Figure 2d; however, this hybrid will be drawn aside under the applied magnetic field of a magnet, indicating the macroscopic characteristic of paramagnetism in the (ZCFO/MNFO)@C-MWCNTs hybrid. 

**Figure 3:**
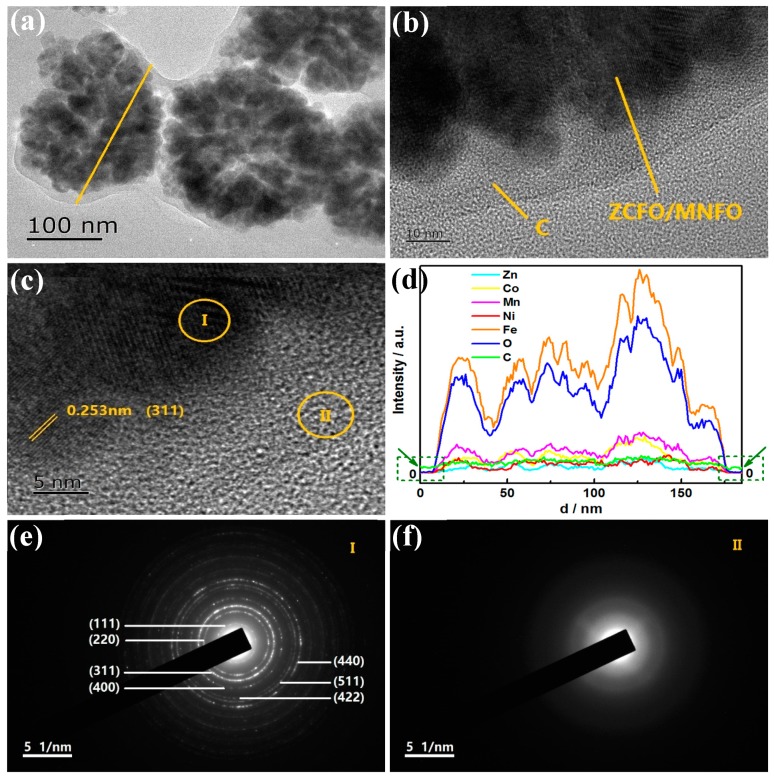
TEM images with different magnifications (**a**) and (**b**), HR-TEM image (**c**), linear distribution of elements (**d**) and SAED patterns in different areas (**e**) and (**f**) of the (ZCFO/MNFO)@C-MWCNTs hybrid.

**Figure 4:**
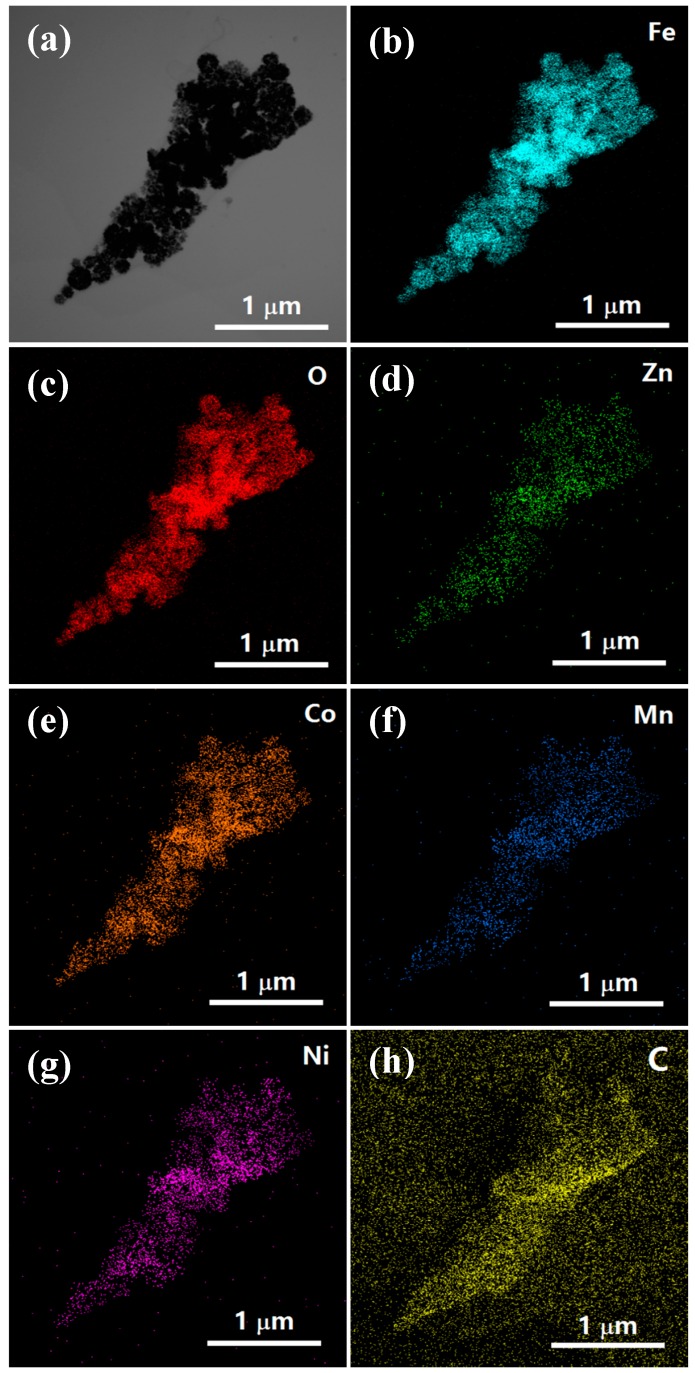
Surface elemental distribution images of the (ZCFO/MNFO)@C-MWCNTs hybrid (**a**) TEM image, (**b**) Fe element, (**c**) O element, (**d**) Zn element, (**e**) Co element, (**f**) Mn element, (**g**) Ni element, (**h**) C element.

Figure 3 shows the transmission electron micrograph of the quaternary hybrid. As shown in Figure 3a, the core of ZCFO/MNFO NPs are formed by many fine micro-grains. Most of the NPs are in the range of 150–230 nm, and some seem to be hollow. Besides, a thin layer of C is coated on the surface of the ZCFO/MNFO NPs and can be clearly seen in Figure 3b; the average thickness of a coated C shell is ~10 nm. The high-resolution transmission electron microscopy image presented in Figure 3c presents clear lattice fringes with spacing of 0.253 nm, accurately displaying the (311) plane of the ZCFO/MNFO core. In addition, the linear distribution of elements on the line displayed in Figure 3a are shown in Figure 3d; it is interesting to note that the Zn, Co, Mn, Ni, Fe, and O elements only exist on the core area of NPs, whose contents are almost close to zero, out of the core area in dashed frames. However, there only exists nonzero content of C element in the shell area, as shown by the arrows, confirming that the shells of NPs are made of carbon, which may be a result of the addition of carbon nanotubes. Moreover, the SAED pattern I of the core area in Figure 3e reveals many diffraction rings corresponding to the (111), (220), (311), (422), (511), and (440) planes of ferrites, further confirming the polycrystalline characteristic of nanoparticles. The SAED pattern II of the shell area displayed in Figure 3f shows the characteristic of an amorphous state in the carbon shell of the NPs. The surface elemental distributions of the (ZCFO/MNFO)@C-MWCNTs hybrid are exhibited in Figure 4; it also indicates that the hybrid consist of Zn, Co, Mn, Ni, Fe, O, and C elements. Except for the C element, the other elements are mainly restricted to the area of ferrite NPs; however, the distribution area of the C element that comes from the MWCNTs is obviously larger than that of the other elements, suggesting a broad distribution of MWCNTs in the hybrid.

**Figure 5:**
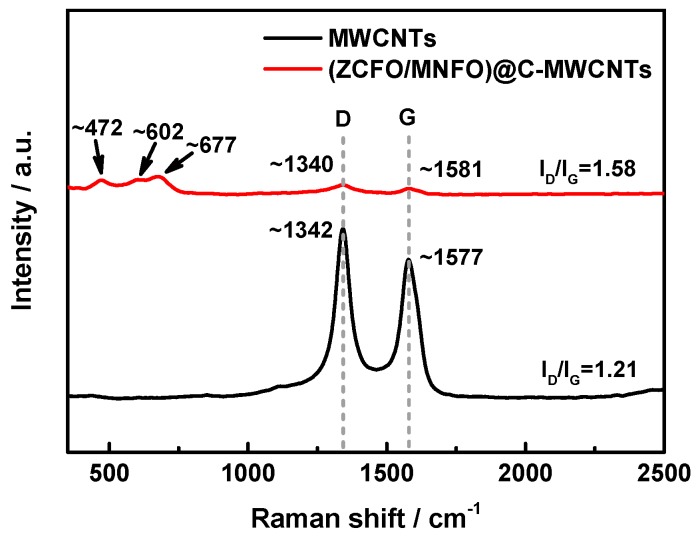
Raman spectrum of the MWCNTs and (ZCFO/MNFO)@C-MWCNTs hybrid.

**Figure 6:**
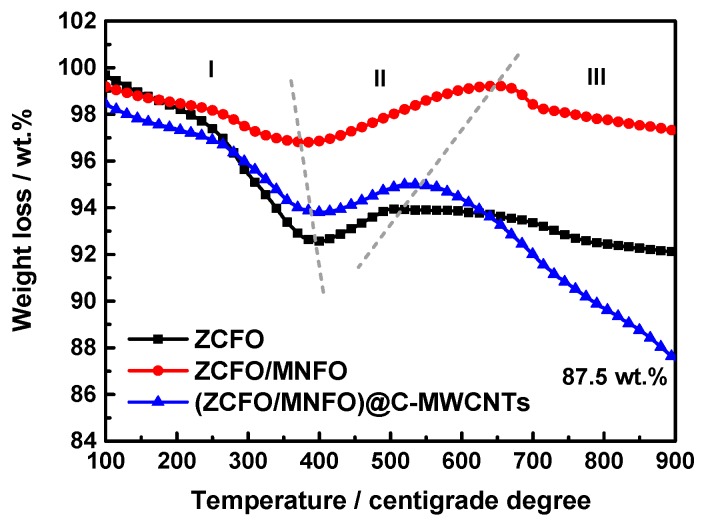
TG curves of ZCFO, ZCFO/MNFO and (ZCFO/MNFO)@C-MWCNTs hybrids.

Raman spectroscopy is an important technology used to characterize the disorder in sp2 carbon materials. Figure 5 presents the Raman spectrum of MWCNTs and the (ZCFO/MNFO)@C-MWCNTs hybrid. It shows that the Raman spectra of pure MWCNTs exhibits two obvious peaks, marked as D band (~1342 cm^−1^) and G band (~1577 cm^−1^), and the intensity ratio of I_D_/I_G_ is 1.21. In general, the D band corresponds to disordered structures and G band relates to the vibration of sp2-bonded carbon atoms. The intensity of D and G band is weakened after the MWCNTs are composited with ferrites; these two bands are located at ~1340 cm^−1^ and ~1581 cm^−1^, respectively. The intensity ratio of I_D_/I_G_ in the quaternary hybrid is 1.58, which is higher than that of raw MWCNTs, indicating a decrease of in-plane sp2 domains’ size and the increase of unrepaired defects with an enhanced degree of disorder [37]. These defects can be served as polarization centers to initiate dipole polarization and related relaxation, which are advantageous to the enhancement of microwave absorption performance of the as-prepared hybrid [38,39]. Moreover, the peaks of Raman shift at around 472 cm^−1^, 602 cm^−1^ and 677 cm^−1^ correspond to the T_2g_, A_1g_ and E_g_ modes of ferrites [36,40]. Usually, the T_2g_ mode represents the characteristic of the octahedral sites, whereas the A_1g_ mode is due to the symmetric stretching of oxygen atoms at the tetrahedral site [41]. Figure 6 displays the TG curves of the ZCFO, ZCFO/MNFO, and (ZCFO/MNFO)@C-MWCNTs hybrids. It can be seen that the weight loss of hybrids during the primary stage is due to the removal of adsorbed water and decomposition of residual organic matter generated from the detergent. The slight weight gain in region II is attributed to the oxidation of ferrites; however, there is a sustained weight loss, as the temperature further increased, which is due to the transformation of ferrites beyond the Curie point and the combustion of MWCNTs. Thus, we can draw a conclusion that the weight percentage of MWCNTs in this quaternary hybrid is ~12 wt%, as expected.

**Figure 7:**
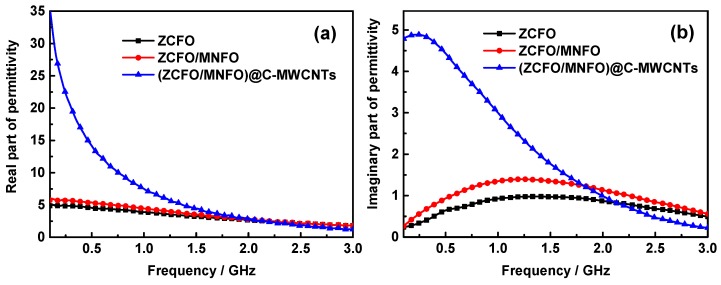

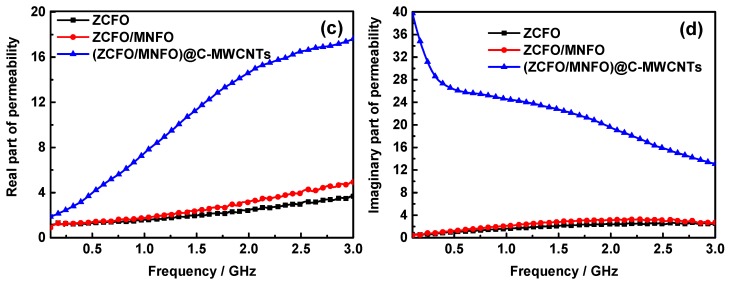
Frequency dependence of the real part of permittivity (**a**), imaginary part of permittivity (**b**), real part of permeability (**c**), and imaginary part of permittivity (**d**).

The complex permittivity (*ε* = *ε*′ − *jε*′′) and permeability (*μ* = *μ*′ − *jμ*′′) of the hybrids are calculated and shown in Figure 7, which are very important in determining the transmission and reflection of electromagnetic waves. As shown in Figure 7a, the real part of permittivity of ZCFO and ZCFO/MNFO are relatively steady; they decrease from 4.88 to 1.82 and 5.75 to 1.83, respectively, when the frequency increased, and the *ε*′ is enhanced slightly in the whole range to coat MNFO on the surface of the ZCFO micro-grains. However, the *ε*′ of the quaternary hybrid was observably strengthened in the low frequency domain due to the addition of MWCNTs; it decreases from 35.36 to 1.22 in the range of 0.1~3 GHz owing to the glorious dielectric properties of MWCNTs [42]. The *ε*′′ of ZCFO increases first and then decreases with the enhancement of frequency in Figure 7b; a peak value of 0.98 at 1.36 GHz can be noted. After cladding, the *ε*′′ of ZCFO/MNFO is reinforced to 1.40 at 1.22 GHz, whereas, this tendency can be further enhanced by the doping of MWCNTs; the imaginary part of permittivity in the quaternary hybrid achieves 4.89 at 0.23 GHz and be comparatively higher in the measurement range, particularly in the low frequency domain. To the best of our knowledge, the permittivity of hybrid in gigahertz band strongly depends on interfacial polarization and dipole polarization. The formation of a NPs@C core-shell structure due to the addition of MWCNTs and the convolving of MWCNTs among particles will immensely increase multiple interfacial polarization; moreover, the defects in imperfect carbon structure and ferrites, and the oxygen-containing groups on MWCNTs will also enhance dipole polarization of the as-prepared quaternary hybrid. Thus, the *ε*′ and *ε*′′ would be enlarged due to the enhancement of dielectric polarization behavior; nevertheless, the generation of displacement current significantly lags behind the build-up potential as the frequency increases, leading to the reduction of both real and imaginary part of permittivity [43,44,45]. Generally speaking, the *ε*′ of MAMs represents the storage capacity of microwave energy during the transmission process of electromagnetic wave, while the *ε*′′ shows the microwave energy loss ability [46]; thus, the relatively higher *ε*′′ in the quaternary hybrid may be helpful for dielectric absorption of the electromagnetic wave. Figure 7c and d show the real and imaginary part of permeability in the as-prepared hybrids. It can be noted that the *μ*′ of ZCFO and ZCFO/MNFO increase with the enhancement of frequency, and their *μ*′′ increase first and then decrease. Whereas, there is a surprise phenomenon that both the *μ*′ and *μ*′′ would be enhanced to a large extent with the cladding of carbon and doping of MWCNTs; the *μ*′ increases from 1.86 to 17.6, while the *μ*′′ decreases from 39.79 to 13.07. This is attributed to that fact that the adhered carbon and MWCNTs can interconnect ferrite NPs in the conductive network, strengthening the coupling effect between different components. As per the theoretical calculation of Kazantseva et al. [47], when the scale of the conductive media is small enough, the effect of conduction currents can be determined by the following dimensionless value:

(1) $$\xi =\frac{\delta R}{\Lambda^{2}},$$

where, *δ* is the scale of conductive media, *R* the radius of ferrite NPs, and Λ can be expressed as:

(2) $$\Lambda =\sqrt{\frac{c^{2}}{2\pi \sigma \left|\omega \right|}},$$

Here, *ω* is angular frequency. Hence, the permeability of the hybrid can be varied with different frequencies for the change of *R* and Λ. This may be the reason why both *μ*′ and *μ*′′ increased with the cladding of carbon and doping of MWCNTs [24]. Overall, the complex formation of multi-phases due to the presence of (ZCFO/MNFO)@C encapsulation structure and MWCNTs is responsible for the reinforcement of both complex permeability and permittivity.

**Figure 8:**
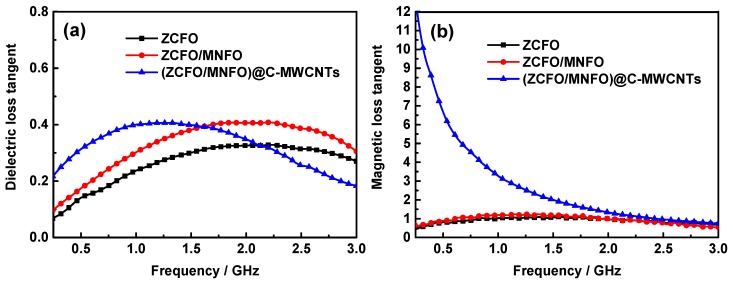
Frequency dependence of dielectric loss tangent (**a**) and magnetic loss tangent (**b**).

Figure 8 displays the magnetic and dielectric loss tangent of the as-prepared hybrids, as shown in Figure 8a. The dielectric loss of ZCFO, ZCFO/MNFO, and (ZCFO/MNFO)@C-MWCNTs increase first and then decrease with enhancement of frequency; the maximum loss value of ZCFO is 0.33 at 2.23 GHz. However, this peak value is enhanced to 0.41 at 2.22 GHz as MNFO is coated on the surface of ZCFO micro-grains owing to the added interface between different phases, which is beneficial in improving interfacial polarization for microwave absorption [48]. It is interesting to note that this dielectric loss peak shifts to a lower frequency of 1.22GHz with the addition of MWCNTs to enhance *ε*′′ in a low frequency domain. Moreover, the magnetic loss tangent of (ZCFO/MNFO)@C-MWCNTs in Figure 8b indicates a strong magnetic loss absorption of microwave, particularly in the low frequency domain; this is mainly due to the increased *μ*′′ of the quaternary hybrid for the reason mentioned above [49]. 

Usually, the electromagnetic absorption of hybrid can be characterized by the parameter of electromagnetic wave reflection loss (*RL*), which is calculated according to the transmission line theory, as follows [50,51]:

(3) $$RL=20\log\left|\frac{Z_{in}-Z_{0}}{Z_{in}+Z_{0}}\right|,$$

The *Z_in_* in Equation (3) is input impedance:

(4) $$Z_{in}=Z_{0}\sqrt{\frac{\mu_{r}}{\varepsilon_{r}}}\tanh\left(j\frac{2\pi fd}{c}\sqrt{\mu_{r}\varepsilon_{r}}\right),$$

**Figure 9:**
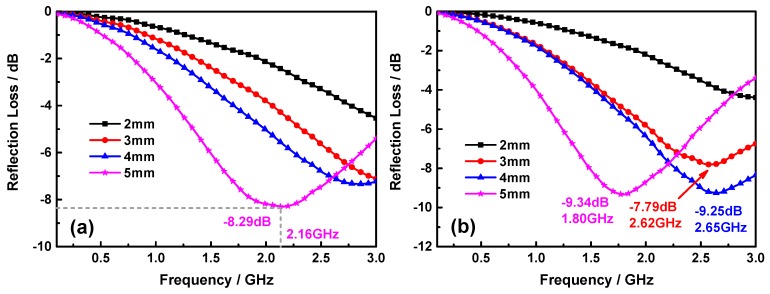

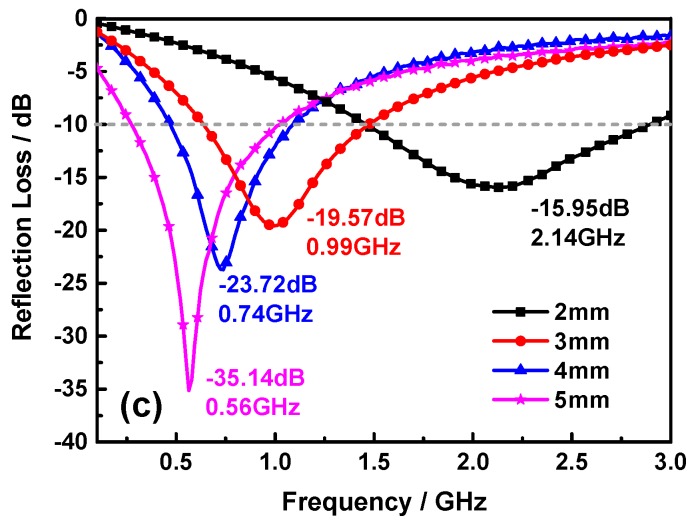
Frequency-dependent *RL* curves of ZCFO (**a**), ZCFO/MNFO (**b**), and (ZCFO/MNFO)@C-MWCNTs (**c**) at different thickness.

Here, *ε_r_* is the permittivity, *μ_r_* the permeability, *Z*_0_ the impedance of air, *d* the thickness of sample, *c* the velocity of light in vacuum, and *f* the frequency. Figure 9 is the *RL* curves of the ZCFO, ZCFO/MNFO, and (ZCFO/MNFO)@C-MWCNTs hybrids at different thickness. As seen in Figure 9a, there is no absorption peak at thickness of 2 mm and maximum *RL* value is −4.54 dB at 3 GHz; this maximum value is enlarged to −7.11 dB when thickness is 3 mm. Nevertheless, there appears an inconspicuous *RL* peak of −7.33 dB at 2.84 GHz at 4 mm, and when the thickness is further increased to 5 mm, the maximum peak shift to 2.16 GHz with intensity of −8.29 dB. Figure 9b shows the *RL* of the ZCFO/MNFO hybrid; it can be noted that the absorption peaks present in the thickness of 3 mm, 4 mm, and 5 mm, and the absorption intensity, are enhanced to −7.79 dB, −9.25 dB, and −9.34 dB, respectively. This is ascribed to the increased interfacial polarization between ZCFO and MNFO. However, this situation will be improved prodigiously as the MWCNTs have been doped in the ferrites to form the quaternary hybrid. It is interesting to note that the maximum *RL* of 2 mm is −15.95 dB at 2.14 GHz with the −10 dB absorption bandwidth between the frequency range of 1.46–2.88 GHz (1.42 GHz), which is attributed to the preferable synergistic effect of dielectric properties of MWCNTs and magnetic properties of ferrites, as well as the enhanced multi-interface polarization and conduction loss of micro-current in MWCNTs [52].These factors are all conducive to improve the electromagnetic absorption performance of the absorber. The electromagnetic wave absorption properties are further enhanced when the thickness of the quaternary hybrid is further increased to 3 mm, 4 mm and 5 mm; the maximum *RL* are −19.57 dB at 0.99 GHz (bandwidth below −10 dB is 0.65–1.46 GHz), −23.72 dB at 0.74 GHz (bandwidth below −10 dB is 0.48–1.09 GHz), and −35.14 dB at 0.56 GHz (bandwidth below −10 dB is 0.27–1.01 GHz), respectively. In general, more than 90% electromagnetic wave energy is dissipated during its transmission in hybrid as the *RL* is below −10 dB, according to the theory of transmission line; here, the maximum absorption peak < −30 dB means that the abrasive electromagnetic wave energy is greater than 99.9% [53]. Moreover, the *RL* absorption peak moves to the lower frequency region with the addition of thickness in the absorber; this is due to the quarter wavelength resonance effect [54]:

(5) $$f=\frac{nc}{4d\sqrt{\left|\mu_{r}\right|\cdot \left|\varepsilon_{r}\right|}},$$

From Equation (5), we can find that the corresponding frequency of the resonance peak will decrease as thickness is increased. 

**Figure 10:**
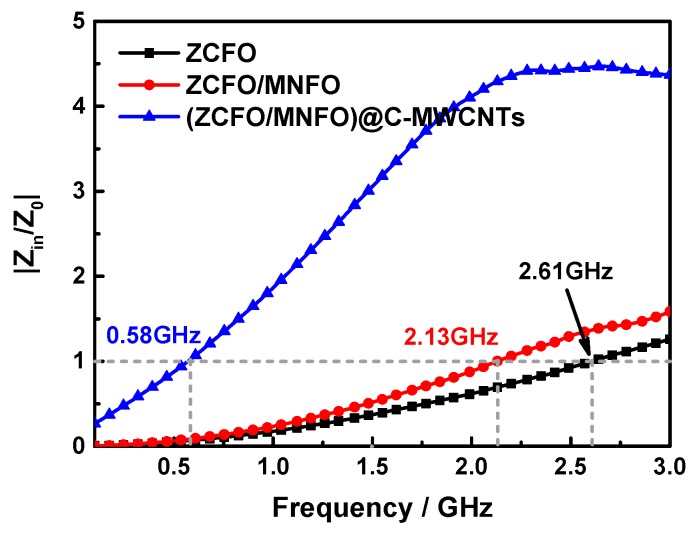
Frequency dependent |Z_in_/Z_0_| values of the ZCFO, ZCFO/MNFO and (ZCFO/MNFO)@C-MWCNTs hybrids at thickness of 5 mm.

The impedance matching condition is very important in determining the incidence and reflection of microwave on the surface of the absorber. The more microwaves enter the absorber to be dissipated when the value of |*Z_in_*/*Z*_0_| is close to one [55]. Figure 10 displays the |*Z_in_*/*Z*_0_| of the ZCFO, ZCFO/MNFO and (ZCFO/MNFO)@C-MWCNTs hybrids at thickness of 5 mm. It suggests that the optimal impedance matching frequency of these three hybrids are 2.61 GHz, 2.13 GHz, and 0.58 GHz, respectively, which is why the frequency of strong absorption peak in the quaternary hybrid is obviously lower than that of the other two, and more electromagnetic wave is transmitted into the hybrid to be dissipated under suitable matching conditions. In addition, the magnetic loss of the ferrite component is always generated from natural resonance loss, eddy current loss, domain wall resonance loss, and hysteresis loss. However, the domain wall resonance loss generally occurs in MHz range and hysteresis loss is weak in the weak field condition [56]. Thus, the eddy current loss and natural resonance loss are the most probable reasons for the magnetic loss in GHz range; here, the eddy current loss is related to the following formula [57]:

(6) $$\mu^{\prime \prime }\approx 2\pi \mu_{0}{\left(\mu^{\prime }\right)}^{2}\sigma d^{2}f/3,$$

Therefore, the characterization parameter of *C*_0_ is calculated as follows [58]:

(7) $$C_{0}=\mu^{\prime \prime }{\left(\mu^{\prime }\right)}^{-2}f^{-1}\approx 2\pi \mu_{0}\sigma d^{2}/3,$$

**Figure 11:**
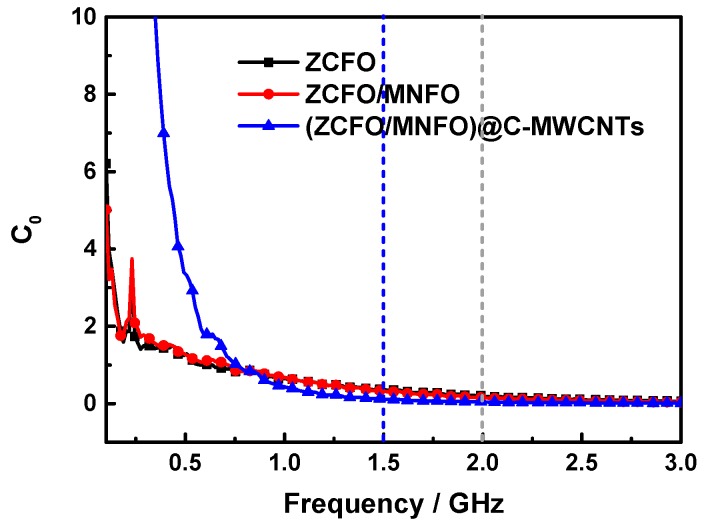
Eddy current loss curves of the ZCFO, ZCFO/MNFO and (ZCFO/MNFO)@C-MWCNTs hybrids.

From Equation (7), we can find that *C*_0_ will maintain a constant as the frequency changes, if the magnetic loss originates from the eddy current loss. Figure 11 presents the eddy current loss curves of the as-prepared hybrids. It can be seen that the *C*_0_ values are almost constant within the frequency range of 2.0–3.0 GHz for ZCFO and ZCFO/MNFO, indicating that the magnetic loss in this range is mainly due to the eddy current loss, while the natural resonance loss mainly exists in the range of 0.1–2.0 GHz. However, this frequency range of the eddy current loss enlarges to 1.5–3.0 GHz for the (ZCFO/MNFO)@C-MWCNTs quaternary hybrid, and the value of *C*_0_ changes dramatically within 0.1–1.5 GHz, manifesting a strong natural resonance loss to enhance the magnetic absorption of the microwave.

The attenuation constant *α* also gives an important meaning to the electromagnetic absorption ability of hybrids; this attenuation property of the absorber can be deduced as [59,60]:

(8) $$\alpha =\frac{\sqrt{2}\pi f}{c}\times \sqrt{(\mu^{\prime \prime }\varepsilon^{\prime \prime }-\mu^{\prime }\varepsilon^{\prime })+\sqrt{{\left(\mu^{\prime \prime }\varepsilon^{\prime \prime }-\mu^{\prime }\varepsilon^{\prime }\right)}^{2}+{\left(\mu^{\prime }\varepsilon^{\prime \prime }+\mu^{\prime \prime }\varepsilon^{\prime }\right)}^{2}}},$$

**Figure 12:**
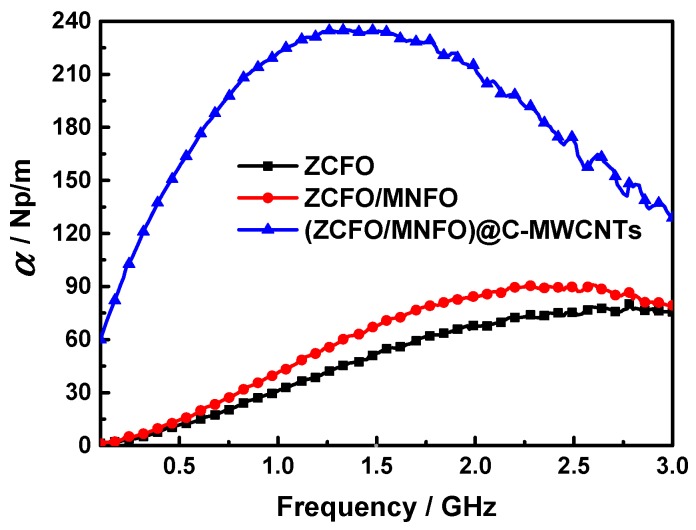
Attenuation constants of the ZCFO, ZCFO/MNFO, and (ZCFO/MNFO)@C-MWCNTs hybrids.

**Figure 13:**
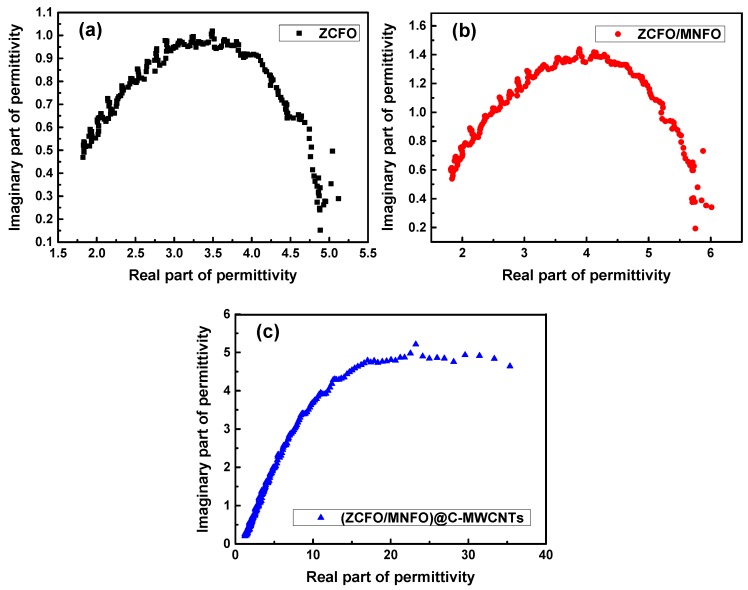
Plots of *ε*′−*ε*′′ for the (**a**) ZCFO, (**b**) ZCFO/MNFO, and (**c**) (ZCFO/MNFO)@C-MWCNTs hybrids.

Figure 12 displays the attenuation constants of the ZCFO, ZCFO/MNFO and (ZCFO/MNFO)@C-MWCNTs hybrids. It shows that the *α* of a single ZCFO is limited and there is a maximum value of 80.14 at 2.78 GHz. This situation can be improved in the ZCFO/MNFO hybrid—the *α* increases first and then decreases in the measurement frequency range; the maximum value is enhanced to 90.78 at 2.26 GHz due to the accessorial interface polarization loss between the ZCFO and MNFO ferrites. However, the attenuation constant in the quaternary hybrid is enhanced to a large extent—it also increases initially and decreases afterwards, while the maximum value of *α* is 235.19 at 1.35 GHz, which is ascribed to the high values of *ε*′′ and *μ*′′, indicating the excellent microwave absorption properties of this hybrid. Furthermore, the plots of *ε*′ and *ε*′′ in the ZCFO, ZCFO/MNFO and (ZCFO/MNFO)@C-MWCNTs hybrids have been displayed in Figure 13. It can be noted that the ε′-ε″ plots present irregular fluctuation, instead of smooth curves, implying the occurrence of interfacial polarization. For the as-prepared ferrite, NPs are constituted by a great deal of micro-grains, which can provide extensive interfaces. Besides, the core-shell structure of the NPs and the entangled state between the NPs and the MWCNTs bring considerable interfacial polarization [28,61]. Another reason is that defects in the carbon-based quaternary hybrid can produce dipole polarization, which is helpful to further enhance the electromagnetic loss [62]. In addition, according to the free electron theory, electron hopping between divalent and trivalent ions as the electromagnetic field is applied may also be an important factor [63,64]. Thus, the electromagnetic absorption performance of this hybrid containing multiple interface polarization, defect dipole polarization, and electron hopping can be greatly reinforced in the low frequency band. 

Table 1 summarizes the electromagnetic wave absorption performances between (ZCFO/MNFO)@C-MWCNTs and related hybrids reported in other literature [65,66,67,68,69,70,71,72,73,74,75]. It is clear that the electromagnetic absorption band of most hybrids are located in the relatively high frequency range. Although the effective absorption bandwidth of the as-synthesized (ZCFO/MNFO)@C-MWCNTs in this work is not wide enough, the hybrid prepared here exhibits superior microwave absorbing performance in low frequency range with an acceptable thickness, which further demonstrates that the (ZCFO/MNFO)@C-MWCNTs is a good candidate for obtaining an enhanced low frequency electromagnetic absorbing property, based on ferrite-carbon nanocomposites.

## 4. Conclusions

In summary, the (Zn_0.5_Co_0.5_Fe_2_O_4_@Mn_0.5_Ni_0.5_Fe_2_O_4_)@C-MWCNTs hybrid with preeminent electromagnetic wave absorption properties in low frequency band was fabricated via a two-step solvothermal synthesis method. The significant electromagnetic absorption properties of the as-prepared paramagnetic quaternary hybrid are attributed to the synergistic effect of dielectric loss (e.g. multiple interface polarization, defects dipole polarization, electron hopping etc.), magnetic loss (e.g. eddy current loss, nature resonance etc.), preferable impedance matching condition, and outstanding attenuation characteristics. Hence, the maximum *RL* of −35.14 dB at 0.56 GHz with an effective absorption bandwidth (*RL* < −10 dB) in the frequency range of 0.27–1.01 GHz (0.74 GHz) can be obtained when the thickness of the absorber is 5 mm. Most important of all, the processing method used here is very simple, and it provides a new route to prepare suitable candidates for the absorption of microwave in low frequency band. 

## Figures and Tables

**Table 1:** Comparison of microwave absorption performance between (ZCFO/MNFO)@C-MWCNTs and related hybrids reported in other literature.

Materials	Thickness	Minimum *RL*	Position	*RL*< −10 dB	Refs.
(ZCFO/MNFO)@C-MWCNTs	5 mm	−35.14 dB	0.56 GHz	0.27–1.01 GHz	This work
CoFe_2_O_4_	2 mm	−55 dB	9.25 GHz	8.2–10.8 GHz	[65]
ZnFe_2_O_4_@rGO@TiO_2_	2.5 mm	−55.6 dB	3.8 GHz	2.8–5.4 GHz	[66]
Fe@ZrO_2_	1.97 mm	−45.36 dB	6.2 GHz	/	[67]
CoFe_2_O_4_/LPA-SWCNT	2 mm	−30.7 dB	12.9 GHz	10.1–17.3 GHz	[68]
CoFe_2_O_4_/NiFe_2_O_4_	4.5 mm	−20.1 dB	9.7 GHz	7.8–16.2 GHz	[69]
SiCnw/GA-S	3.63 mm	−54.8 dB	5.3 GHz	/	[70]
CoFe_2_O_4_/rGO@PVP	1.96 mm	−56.8 dB	15.7 GHz	10.64–17.44 GHz	[71]
Ni_0.6_Zn_0.3_Mn_0.1_Fe_2_O_4_	10 mm	-20 dB	1.8 GHz	/	[72]
MWCNTs/Co-Ni/Fe_3_O_4_	3 mm	−13.57 dB	1.51 GHz	/	[73]
SnS/SnO_2_@C-16V	5 mm	< −12 dB	/	1.5–2 GHz	[74]
Fe@Fe_3_C@C	2.4 mm	−58.0 dB	8.68 GHz	/	[75]
